# Supplementary material for: Heparin-based hydrogel scaffolding alters the transcriptomic profile and increases the chemoresistance of MDA-MB-231 triple-negative breast cancer cells
Source: Biomater Sci. 2020 Feb 13;8(10):2786–96. doi: 10.1039/c9bm01481k (PMC7497406; doi:10.1039/c9bm01481k)
Supplement: Supplementary file 2 [file BM-008-C9BM01481K-s002.zip › Supplementary File 4/HP-B hydrogel + EGF (s)vControl/Pathways/my_analysis.Gsea.1545201170987/HALLMARK_UNFOLDED_PROTEIN_RESPONSE.html]

Details for gene set HALLMARK\_UNFOLDED\_PROTEIN\_RESPONSE[GSEA]

|  || Dataset | expr.class.cls#HYDEGF\_versus\_CONTROL.class.cls#HYDEGF\_versus\_CONTROL\_repos |
| Phenotype | class.cls#HYDEGF\_versus\_CONTROL\_repos |
| Upregulated in class | CONTROL |
| GeneSet | HALLMARK\_UNFOLDED\_PROTEIN\_RESPONSE |
| Enrichment Score (ES) | -0.2264667 |
| Normalized Enrichment Score (NES) | -0.99022174 |
| Nominal p-value | 0.4240196 |
| FDR q-value | 0.5330383 |
| FWER p-Value | 1.0 |
Table: GSEA Results Summary

  

Fig 1: Enrichment plot: HALLMARK\_UNFOLDED\_PROTEIN\_RESPONSE      
 Profile of the Running ES Score & Positions of GeneSet Members on the Rank Ordered List

  

| PROBE | DESCRIPTION (from dataset) | GENE SYMBOL | GENE\_TITLE | RANK IN GENE LIST | RANK METRIC SCORE | RUNNING ES | CORE ENRICHMENT || 1 | SLC7A5 | na |  |  | 239 | 2.501 | 0.0192 | No |
| 2 | ATF3 | na |  |  | 473 | 2.154 | 0.0342 | No |
| 3 | TATDN2 | na |  |  | 485 | 2.141 | 0.0608 | No |
| 4 | NOP56 | na |  |  | 837 | 1.790 | 0.0651 | No |
| 5 | EXOSC2 | na |  |  | 960 | 1.714 | 0.0804 | No |
| 6 | NOP14 | na |  |  | 1213 | 1.565 | 0.0870 | No |
| 7 | ASNS | na |  |  | 1455 | 1.448 | 0.0927 | No |
| 8 | CXXC1 | na |  |  | 1507 | 1.431 | 0.1082 | No |
| 9 | GOSR2 | na |  |  | 1820 | 1.319 | 0.1085 | No |
| 10 | EXOSC9 | na |  |  | 2116 | 1.234 | 0.1087 | No |
| 11 | EIF4A3 | na |  |  | 2321 | 1.172 | 0.1129 | No |
| 12 | EXOSC10 | na |  |  | 2325 | 1.170 | 0.1275 | No |
| 13 | ZBTB17 | na |  |  | 2445 | 1.142 | 0.1358 | No |
| 14 | EIF4A1 | na |  |  | 2683 | 1.084 | 0.1371 | No |
| 15 | CEBPG | na |  |  | 2757 | 1.062 | 0.1467 | No |
| 16 | ARFGAP1 | na |  |  | 2821 | 1.048 | 0.1567 | No |
| 17 | STC2 | na |  |  | 3009 | 1.002 | 0.1596 | No |
| 18 | ATF4 | na |  |  | 3079 | 0.986 | 0.1684 | No |
| 19 | RRP9 | na |  |  | 3207 | 0.957 | 0.1739 | No |
| 20 | SLC1A4 | na |  |  | 3358 | 0.931 | 0.1778 | No |
| 21 | TSPYL2 | na |  |  | 3370 | 0.928 | 0.1890 | No |
| 22 | H2AFX | na |  |  | 3433 | 0.916 | 0.1974 | No |
| 23 | EDC4 | na |  |  | 4239 | 0.776 | 0.1650 | No |
| 24 | PDIA5 | na |  |  | 4960 | 0.658 | 0.1357 | No |
| 25 | PSAT1 | na |  |  | 5076 | 0.641 | 0.1378 | No |
| 26 | IARS | na |  |  | 5493 | 0.577 | 0.1233 | No |
| 27 | NOLC1 | na |  |  | 5664 | 0.551 | 0.1213 | No |
| 28 | GEMIN4 | na |  |  | 6060 | 0.495 | 0.1069 | No |
| 29 | KHSRP | na |  |  | 6494 | 0.437 | 0.0898 | No |
| 30 | PREB | na |  |  | 7385 | 0.319 | 0.0472 | No |
| 31 | LSM1 | na |  |  | 7620 | 0.286 | 0.0386 | No |
| 32 | WFS1 | na |  |  | 7951 | 0.249 | 0.0245 | No |
| 33 | SDAD1 | na |  |  | 8181 | 0.228 | 0.0153 | No |
| 34 | FUS | na |  |  | 8347 | 0.202 | 0.0093 | No |
| 35 | EXOSC4 | na |  |  | 8453 | 0.195 | 0.0062 | No |
| 36 | DCTN1 | na |  |  | 8546 | 0.182 | 0.0037 | No |
| 37 | LSM4 | na |  |  | 8591 | 0.175 | 0.0036 | No |
| 38 | MTHFD2 | na |  |  | 8784 | 0.152 | -0.0045 | No |
| 39 | SHC1 | na |  |  | 8800 | 0.150 | -0.0034 | No |
| 40 | TARS | na |  |  | 8852 | 0.141 | -0.0043 | No |
| 41 | EXOC2 | na |  |  | 8926 | 0.131 | -0.0064 | No |
| 42 | DKC1 | na |  |  | 9375 | 0.079 | -0.0289 | No |
| 43 | EIF4EBP1 | na |  |  | 9493 | 0.076 | -0.0341 | No |
| 44 | PARN | na |  |  | 9684 | 0.051 | -0.0433 | No |
| 45 | EXOSC5 | na |  |  | 10294 | -0.021 | -0.0750 | No |
| 46 | EIF4A2 | na |  |  | 10318 | -0.025 | -0.0759 | No |
| 47 | DCP2 | na |  |  | 10320 | -0.026 | -0.0756 | No |
| 48 | CEBPB | na |  |  | 10406 | -0.036 | -0.0796 | No |
| 49 | TUBB2A | na |  |  | 10631 | -0.057 | -0.0906 | No |
| 50 | CNOT4 | na |  |  | 10700 | -0.065 | -0.0933 | No |
| 51 | ERN1 | na |  |  | 10724 | -0.068 | -0.0937 | No |
| 52 | CHAC1 | na |  |  | 10835 | -0.084 | -0.0984 | No |
| 53 | EIF2AK3 | na |  |  | 10898 | -0.095 | -0.1004 | No |
| 54 | KIF5B | na |  |  | 11200 | -0.123 | -0.1147 | No |
| 55 | FKBP14 | na |  |  | 11253 | -0.130 | -0.1157 | No |
| 56 | NFYB | na |  |  | 11392 | -0.146 | -0.1211 | No |
| 57 | EXOSC1 | na |  |  | 11497 | -0.163 | -0.1245 | No |
| 58 | DDX10 | na |  |  | 11703 | -0.187 | -0.1329 | No |
| 59 | BAG3 | na |  |  | 11755 | -0.196 | -0.1330 | No |
| 60 | NFYA | na |  |  | 11800 | -0.202 | -0.1328 | No |
| 61 | DNAJA4 | na |  |  | 11944 | -0.214 | -0.1376 | No |
| 62 | XPOT | na |  |  | 12013 | -0.218 | -0.1384 | No |
| 63 | IFIT1 | na |  |  | 12079 | -0.229 | -0.1389 | No |
| 64 | SKIV2L2 | na |  |  | 12709 | -0.315 | -0.1678 | No |
| 65 | HSPA9 | na |  |  | 13160 | -0.362 | -0.1868 | No |
| 66 | TTC37 | na |  |  | 13249 | -0.377 | -0.1866 | No |
| 67 | WIPI1 | na |  |  | 13313 | -0.388 | -0.1850 | No |
| 68 | NHP2 | na |  |  | 13491 | -0.412 | -0.1891 | No |
| 69 | HYOU1 | na |  |  | 13500 | -0.413 | -0.1843 | No |
| 70 | SLC30A5 | na |  |  | 13943 | -0.478 | -0.2013 | No |
| 71 | BANF1 | na |  |  | 14157 | -0.500 | -0.2062 | No |
| 72 | CCL2 | na |  |  | 14503 | -0.557 | -0.2172 | No |
| 73 | SRPRB | na |  |  | 14628 | -0.577 | -0.2164 | No |
| 74 | CKS1B | na |  |  | 14664 | -0.584 | -0.2108 | No |
| 75 | CNOT6 | na |  |  | 14683 | -0.588 | -0.2043 | No |
| 76 | YIF1A | na |  |  | 15107 | -0.654 | -0.2182 | Yes |
| 77 | ALDH18A1 | na |  |  | 15175 | -0.665 | -0.2133 | Yes |
| 78 | ATP6V0D1 | na |  |  | 15249 | -0.678 | -0.2085 | Yes |
| 79 | SPCS3 | na |  |  | 15475 | -0.716 | -0.2112 | Yes |
| 80 | CNOT2 | na |  |  | 15547 | -0.732 | -0.2057 | Yes |
| 81 | SEC11A | na |  |  | 15723 | -0.769 | -0.2051 | Yes |
| 82 | SERP1 | na |  |  | 15766 | -0.778 | -0.1974 | Yes |
| 83 | XBP1 | na |  |  | 16049 | -0.833 | -0.2017 | Yes |
| 84 | DCP1A | na |  |  | 16060 | -0.836 | -0.1916 | Yes |
| 85 | IGFBP1 | na |  |  | 16061 | -0.836 | -0.1810 | Yes |
| 86 | SEC31A | na |  |  | 16110 | -0.846 | -0.1728 | Yes |
| 87 | IMP3 | na |  |  | 16202 | -0.870 | -0.1666 | Yes |
| 88 | DDIT4 | na |  |  | 16203 | -0.871 | -0.1555 | Yes |
| 89 | EIF2S1 | na |  |  | 16348 | -0.909 | -0.1516 | Yes |
| 90 | ATF6 | na |  |  | 16471 | -0.939 | -0.1460 | Yes |
| 91 | PAIP1 | na |  |  | 16664 | -0.990 | -0.1436 | Yes |
| 92 | POP4 | na |  |  | 16793 | -1.024 | -0.1373 | Yes |
| 93 | EDEM1 | na |  |  | 17083 | -1.110 | -0.1384 | Yes |
| 94 | RPS14 | na |  |  | 17145 | -1.127 | -0.1273 | Yes |
| 95 | EIF4E | na |  |  | 17315 | -1.190 | -0.1211 | Yes |
| 96 | HERPUD1 | na |  |  | 17426 | -1.226 | -0.1113 | Yes |
| 97 | DNAJB9 | na |  |  | 17463 | -1.242 | -0.0975 | Yes |
| 98 | PDIA6 | na |  |  | 17542 | -1.274 | -0.0854 | Yes |
| 99 | CALR | na |  |  | 17688 | -1.326 | -0.0762 | Yes |
| 100 | HSPA5 | na |  |  | 17766 | -1.367 | -0.0629 | Yes |
| 101 | SPCS1 | na |  |  | 18510 | -1.845 | -0.0785 | Yes |
| 102 | SSR1 | na |  |  | 18693 | -2.038 | -0.0622 | Yes |
| 103 | DNAJC3 | na |  |  | 18716 | -2.072 | -0.0371 | Yes |
| 104 | KDELR3 | na |  |  | 18881 | -2.328 | -0.0162 | Yes |
| 105 | HSP90B1 | na |  |  | 19003 | -2.588 | 0.0102 | Yes |
Table: GSEA details [plain text format]

  

Fig 2: HALLMARK\_UNFOLDED\_PROTEIN\_RESPONSE      
 Blue-Pink O' Gram in the Space of the Analyzed GeneSet

  

Fig 3: HALLMARK\_UNFOLDED\_PROTEIN\_RESPONSE: Random ES distribution      
 Gene set null distribution of ES for **HALLMARK\_UNFOLDED\_PROTEIN\_RESPONSE**
